# Glomerular injury induced by vinyl carbamate in A/J inbred mice: a novel model of membranoproliferative glomerulonephritis

**DOI:** 10.3389/fphar.2024.1462936

**Published:** 2024-09-06

**Authors:** Athena Y. Gong, Ying Jin Qiao, Mengxuan Chen, Zubia Alam, Deepak K. Malhotra, Lance Dworkin, Wenjun Ju, William T. Gunning

**Affiliations:** ^1^ College of Literature, Science and the Arts, University of Michigan, Ann Arbor, MI, United States; ^2^ Michigan O’Brien Kidney National Resource Center, Department of Internal Medicine, University of Michigan, Ann Arbor, MI, United States; ^3^ Department of Medicine, University of Toledo College of Medicine, Toledo, OH, United States; ^4^ Department of Pathology, Medical College of Ohio at University of Toledo, Toledo, OH, United States

**Keywords:** complement, C3 glomerulonephritis, C3 glomerulopathy, glomerular endothelial cells, dense deposit disease

## Abstract

Ethyl carbamate (EC) is a process contaminant found in fermented foods and alcoholic beverages. Metabolic conversion of ethyl carbamate generates vinyl carbamate (VC), a carcinogenic metabolite. EC, as a Group 2A probable human carcinogen, and the more potent VC, are known to cause tumors in rodents. However, their effects on the kidney are unknown and were explored here. Female A/J inbred mice received an intraperitoneal injection of vehicle or VC. Beginning 5 weeks after VC injection, mice showed signs of moribund state. Mouse necropsies revealed renal glomerular injury that histopathologically recapitulated human membranoproliferative glomerulonephritis (MPGN), as evidenced by light microscopy, immunostaining for immunoglobulins and complements, and electron microscopy. To determine the molecular pathomechanisms, a *post-hoc* analysis was performed on a publicly available RNA-Seq transcriptome of kidneys from control rats and rats treated with fermented wine containing high concentrations of EC. Kyoto Encyclopedia of Genes and Genomes pathway enrichment analyses of the differentially expressed genes revealed that the complement and coagulation cascades were a top predicted biological process involved. Furthermore, pathway-based data integration and visualization revealed that key regulators of complement activation were altered by high EC treatment. Among these, complement factors (CF) D and H, critical positive and negative regulators of the alternative pathway, respectively, were most affected, with CFD induced by 3.49-fold and CFH repressed by 5.9-fold, underscoring a hyperactive alternative pathway. Consistently, exposure of primary glomerular endothelial cells to EC or VC resulted in induction of CFD and repression of CFH, accompanied by increased fixation of C3 and C5b9. This effect seems to be mediated by Ras, one of the top genes that interact with both EC and VC, as identified by analyzing the chemical-gene/protein interactions database. Indeed, EC or VC-elicited complement activation was associated with activation of Ras signaling, but was abolished by the Ras inhibitor farnesyl thiosalicylic acid. Collectively, our findings suggest that VC, a metabolite of EC, induces glomerular injury in mice akin to human MPGN, possibly via perturbing the expression of complement regulators, resulting in an effect that favors activation of the alternative complement pathway.

## Introduction

Membranoproliferative glomerulonephritis (MPGN) is an uncommon pattern of kidney injury caused by aberrant immune responses that primarily affect renal glomeruli (Sethi and Fervenza, 2012). Microscopically, MPGN is characterized by typical lesions in glomeruli, including diffuse proliferation of mesangial and endocapillary cells, which often leads to a lobular appearance of the glomerular tuft. Additionally, there is thickening of glomerular capillary walls due to deposition of immune complexes and/or complement factors, interposition of the mesangial cells and other cellular elements between the glomerular basement membrane (GBM) and the endothelial cells, and new basement membrane formation (Fogo et al., 2015; Cook and Pickering, 2015). As a histologically defined diagnosis, MPGN does not represent a single disease entity but is seen in a variety of disease processes that may share common pathogenetic mechanisms (Noris et al., 2023). Thus, depending on the etiology, MPGN could be subdivided into the idiopathic form and the secondary form (Sethi and Fervenza, 2012; Cook and Pickering, 2015). While idiopathic MPGN is histologically featured by isolated or predominant C3 fixation in glomeruli on immunofluorescent staining, secondary MPGN, usually marked by additional immune complex deposition in glomeruli, is associated with several systemic diseases including chronic infectious diseases, autoimmune conditions like systemic lupus erythematosus, and hematological disorders. Clinically, MPGN manifests as hematuria, heavy proteinuria, nephrotic syndrome, and associated complications. Patients with MPGN tend to have a protracted clinical course with repeated disease flares and progress to end-stage renal failure in approximately 20%–50% of patients depending on the cohorts studied (Sethi and Fervenza, 2012). To date, MPGN remains a therapeutic challenge for clinical practice with no definitive treatments available. To develop novel therapies and improve the clinical outcomes, it is imperative to understand the pathogenic mechanism of MPGN and identify novel therapeutic targets (Noris et al., 2023). Unfortunately, despite a well-established histomorphologic pattern of pathologic changes, the molecular pathomechanisms of MPGN are poorly understood. This knowledge gap is due in large part to the lack of a pragmatic preclinical disease model for MPGN (Vernon et al., 2011).

Ethyl carbamate (EC), also known as urethane (an ester of carbamic acid), is a process contaminant and harmful by-product naturally formed in fermented foods and alcoholic beverages during the fermentation or storage (Gowd et al., 2018; Shalamitskiy et al., 2023). When metabolized by cytochrome P-450 enzymes in the liver, lung, or other organs, EC converts to vinyl carbamate (VC), a carcinogenic metabolite (Forkert et al., 2001; Guengerich and Kim, 1991). As a Group 2A probable human carcinogen, EC, along with the more potent VC, has been shown to induce tumors in rodents in the lung and other organs (Forkert, 2010; Hernandez and Forkert, 2007). In humans, burgeoning evidence links the consumption of ultra-processed foods, including fermented foods, to the prevalence of kidney disease. For instance, Kityo and Lee Kityo and Lee (2022) found that intake of ultra-processed foods was associated with high prevalence of chronic kidney disease and reduced kidney function. However, the exact effect of EC or VC on the kidney is unknown. Here, we report our serendipitous discovery of an MPGN-like renal pathology associated with isolated C3 glomerular deposition in VC-treated mice. To elucidate the underlying pathomechanisms, in-depth analysis of the publicly available renal transcriptome derived from EC-treated rats (Wang et al., 2021), combined with validation studies in glomerular endothelial cells, revealed that VC and EC are capable of activating the alternative complement pathway. This VC-induced mouse model offers a valuable tool for further research into MPGN pathogenesis and potential therapies.

## Materials and methods

### Animal experimental design

Animal experiments were approved by the Institutional Animal Care and Use Committee (IACUC) of the Medical College of Ohio at University of Toledo, and they conform to US Department of Agriculture regulations and the National Institutes of Health guidelines for humane care and use of laboratory animals. Female A/J inbred mice at 5–6°weeks old were purchased from the Jackson Laboratory (Bar Harbor, ME). Mice were housed in a 12 h light/dark cycle at a temperature of 72°F ± 2°F and relative humidity of 40%–60% with *ad libitum* access to regular chow (rodent diet AIN-76A) and drinking water. After acclimation for 2 weeks, mice were randomized to receive a single intraperitoneal injection of VC (Toronto Research Chemicals, North York, Ontario, Canada) in 0.2 mL of saline at a dose of 60 mg/kg/body *wt*, a dose established in the literature and as previously used (Gunning et al., 2002). Mice were scheduled to be euthanized at various time points beginning at 5 weeks through 24 weeks post injection. Control mice received a single intraperitoneal injection of an equal volume of saline. As a chemopreventive intervention, a subgroup of mice received the chemopreventive agent myo-inositol (10.0 g/kg/diet, Sigma, St. Louis, MO, United States) in diet starting 1 week before or dexamethasone (100 mg/kg/diet, Sigma, St. Louis, MO, United States) in diet starting 1 week after the administration of VC and continuing in the diet for the duration of the experiment as described previously (Gunning et al., 2000). Mice were evaluated weekly throughout the experiments. Upon euthanasia, kidneys were excised and harvested. One portion of the kidney was fixed in 10% phosphate-buffered formalin for morphologic analyses, and another part was embedded in OCT and snap frozen immediately in liquid nitrogen for subsequent fluorescence immunohistochemistry staining. Cortical specimens were processed for transmission electron microscopy (EM).

### Histopathology assessment

Formalin-fixed mouse kidneys were prepared in 3-μm-thick sections. For general histology, sections were processed for hematoxylin and eosin staining by routine procedures. In brief, after deparaffinization through xylene and graded alcohols, sections were rehydrated with distilled water. Subsequently sections were subjected to hematoxylin stain, followed by eosin counterstain. Then, sections were dehydrated through graded alcohols, cleared in 3 changes of xylene, and mounted using a drop of mounting medium and a coverslip. Mouse kidney histology was visualized using a light microscope and evaluated in a blinded manner.

### Transmission electron microscopy

Kidney cortical tissues were cut into small pieces (1 mm^3^), fixed with 2.5% glutaraldehyde, and embedded in Epon 812 (Polysciences Inc., Warrington, PA). Conventional electron micrographs were obtained using the Philips CM 10 transmission electron microscope (Philips Electronics, Eindhoven, Netherlands) or the Talos L120C transmission electron microscope (Thermo Fisher Scientific, Waltham, MA, United States) operated at 80 kV. Samples were evaluated in a blinded manner by an investigator.

### Glomerular isolation and primary culture of glomerular endothelial cells

Isolation of mouse glomeruli and preparation of primary glomerular endothelial cells were performed as described previously (Nagao et al., 2007), with minor modifications. In essence, mice were euthanized, and kidneys were perfused with 5 mL of phosphate-buffered saline containing magnetic iron oxide particles (Sigma). The magnetic particles-based glomerular isolation was performed in a ventilated dissection hood under clean-contaminated condition via an approach using a magnetic particle concentrator. Isolated glomeruli were pooled and washed twice by brief centrifugation and then digested in 1 mg/mL collagenase (Sigma) for 30 min at 37°C with occasional vortexing. After brief centrifugation to remove undigested tissue chunks, the supernatant containing single-cell suspension of glomerular endothelial cells was collected, spun, and resuspended in endothelial culture medium supplemented with 20% heat-inactivated fetal bovine serum (FBS) as used before with some modifications (Leopold et al., 2019; Colvert et al., 2023). Cells were plated at a density of 4.5 × 10^5^ cells on collagen-coated 35 mm petri dishes. Small colonies of mouse glomerular endothelial cells were observed within 7–10 days after plating. Contaminating mesangial and epithelial cells were removed by sequential trypsinization until a culture with over 90% endothelial cell purity was achieved, based on characterization by the expression of endothelial-specific markers, such as PECAM1. Mouse glomerular endothelial cells were maintained in the aforementioned growth medium and used in subsequent experiments.

### Cell culture experiments

Primary glomerular endothelial cells were cultured in normal growth medium until subconfluence. Then cultured endothelial cells underwent serum starvation overnight in serum-reduced endothelial culture medium containing 5% FBS. Subsequently, VC (0.625 mM) (Li et al., 2020), EC (25 mM) (Cui et al., 2016) or vehicle was added to the culture in the presence or absence of the Ras inhibitor farnesyl thiosalicylic acid (100 µM) (Wu et al., 2019) or vehicle for 24 h. The doses of various drugs were adopted based on previous studies and confirmed by tetrazolium-based MTT assay in pilot experiments to barely affect cellular viability. After treatments, cells were fixed or cell lysates were collected for further examinations.

### Fluorescent immunohistochemistry staining

Cultured cells or cryosections of cortical kidney specimens were fixed with 4% paraformaldehyde, permeabilized using 0.1% Triton X-100, and incubated with the primary antibody against C3 (Santa Cruz Biotechnology, Dallas, TX, United States) or C5b9 (Santa Cruz Biotechnology) overnight, followed by Alexa fluorophore-conjugated secondary antibody staining (Thermo Fisher Scientific). Cells or sections were subsequently washed with PBS and counterstained with 4′, 6-diamidino-2-phenylindole (DAPI, Abcam, San Francisco, CA, United States). Finally, sections were visualized using a fluorescence microscope. As negative controls, specimens were subjected to the same procedures except that primary antibodies were replaced with nonimmune IgG, and no specific staining was noted.

### Western immunoblot analysis

Cultured cells were lysed in radioimmunoprecipitation (RIPA) buffer containing protease inhibitors. Protein samples were processed for immunoblot analysis as previously described. The antibodies against complement factor (CF) D, CFH, Raf, glycogen synthase kinase (GSK) 3β, p-GSK3β^S9^ and GAPDH were purchased from Santa Cruz Biotechnology, the antibody against p-Raf was purchased from Cell Signaling Technology (Danvers, MA, United States).

### Bioinformatics analysis

A publicly available kidney and liver RNA-sequencing transcriptomic dataset was retrieved from the Wang study (Wang et al., 2021), in which rats were treated daily with fermented wine containing a high concentration of EC (10 mg/kg) for 1 week. Differentially expressed genes (DEGs) in the kidney or liver between high EC-treated rats and control rats with statistical significance were identified. To discover the significantly altered pathways involving selected DEGs, pathway enrichment analysis was performed based on Kyoto Encyclopedia of Genes and Genomes (KEGG), which is a knowledge base for the systematic analysis of gene functions in terms of networks of genes and molecules. The DEGs were further subjected to pathway enrichment analysis based on the WikiPathways database, which is continuously updated and community curated pathway database dedicated to open science and captures the collective knowledge represented in biological pathways (Slenter et al., 2018). Additional pathway enrichment analysis was performed based on the Rat Genome Database (RGD), a cross-species knowledgebase and the premier online resource for rat genetic and physiologic data (Kaldunski et al., 2022). Moreover, to examine gene-disease relationships, DEGs with statistical significance were subjected to enrichment analysis based on Disease Ontology (DO), which is a database describing human gene function and disease that can be used to consider the interactions and functions of DEGs with disease. In this study, KEGG, WikiPathways, RGD, and DO analyses of DEGs were performed by ShinyGo, a graphical gene-set enrichment tool. To quantify the most important functional pathways, *P* values of 0.05 was employed as standardized metrics. Furthermore, genes enriched in the complement and coagulation cascades were subjected to hierarchical cluster analysis and heat map representation by using Tbtools.

In addition, to explore the possible molecular mechanism underlying EC or VC-induced glomerular injury, we further accessed and analyzed the Comparative Toxicogenomics Database (http://ctdbase.org), which harmonizes cross-species heterogeneous data for chemical exposures and their biological repercussions by manually curating and interrelating chemical, gene, phenotype, anatomy, disease, taxa, and exposure content from the published literature (Davis et al., 2023). The top interacting genes for EC or VC were revealed and results were compared to find the common interacting genes that interact with both EC and VC for the subsequent mechanistic experiments.

### Statistical analyses

All data are expressed as mean ± SD. Unless otherwise indicated, all *in vitro* experimental observations were repeated 3 times. For immunoblot analysis, bands were scanned and the integrated pixel density was determined using a densitometer and analyzed by the ImageJ program (version 1.48 National Institutes of Health, Bethesda, MD, United States). Statistical analysis of the data from multiple groups was performed by one-way ANOVA tests followed by Tukey tests. Data from two groups were compared by using a two-sided *t*-test. Statistical analyses were performed using GraphPad Prism 8.0 software (GraphPad Software, San Diego, CA) or SPSS 22 (IBM Corporation, Armonk, NY). *P* < 0.05 was considered statistically significant.

## Results

### VC, the EC metabolite, elicits glomerular injury in A/J mice with histopathological changes highly reminiscent of the pathology of MPGN

**FIGURE 1:**
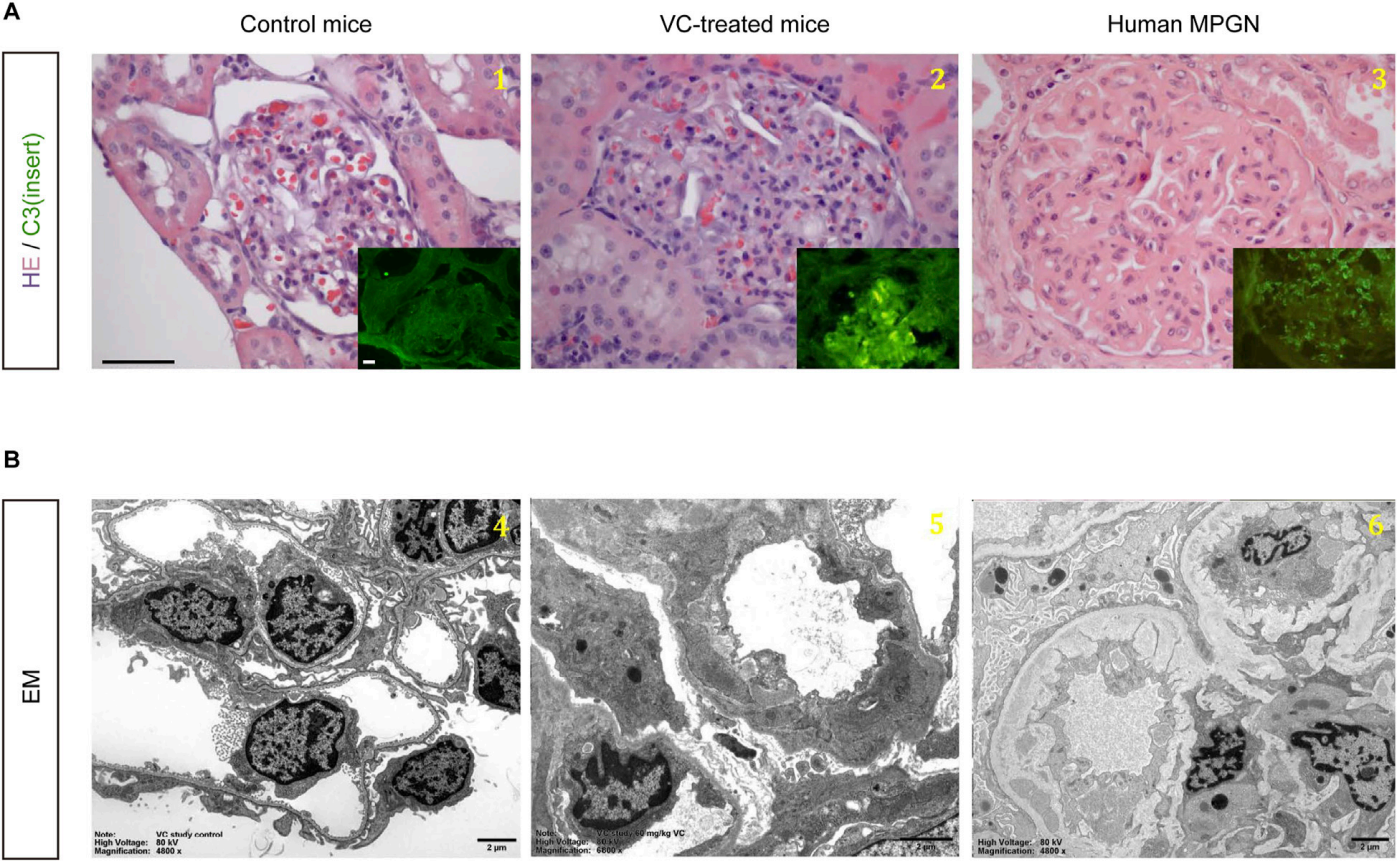
VC treatment causes MPGN-like renal glomerular changes in A/J mice. **(A)** Representative micrographs show hematoxylin and eosin staining (Scale bar, 50 μm) and immunofluorescent staining for C3 (Scale bar, 10 μm) in kidney specimens collected from control mice and vinyl carbamate (VC)-treated mice. As a comparison, micrographs of typical lesions of human membranoproliferative glomerulonephritis (MPGN) are shown in parallel. **(B)** Representative electron micrographs show transmission electron microscopy (EM) of kidney specimens collected from control mice and VC-treated mice (Scale bar, 2 μm). As a comparison, EM of typical lesions of human MPGN is shown in parallel. (1) and (4): control mice; (2) and (5): VC-treated mice; (3) and (6): human MPGN.

At 5 weeks after VC injection, mice had evidence of a moribund state, suggesting that VC, at the dose of 60 mg/kg, was toxic. By 12 weeks after VC injection, 97 of 240 VC-treated mice (40%) had died. Moribund mice were euthanized in order to investigate the cause of death of other mice. It was determined that VC-treated animals likely died from azotemia or renal failure. While the kidneys of one untreated control mouse (#2), which did not receive VC, were normal, all 14 mice necropsied that received VC with or without *myo*-inositol or dexamethasone co-treatment were found to have glomerular injury, the likely cause of their moribund state (Supplementary Table 1). Hematoxylin and eosin staining under light microscopy revealed that glomerular injury in VC-treated mice was characterized by prominent lobularization of the glomeruli, mesangial hypercellularity and expansion, segmental endocapillary proliferation, neutrophil infiltration, and thickening of the capillary walls. (Figure 1A). On immunofluorescent staining, isolated and granular C3 staining was noted in mesangium along with coarse linear capillary staining, whilst immunoglobulin staining was negative (data not shown). This is consistent with EM findings, where granular formless deposits were found in the mesangium and subendothelium and were not electron dense in character. EM also demonstrated interposed cells along the glomerular basement membrane, new basement membrane material between the interposed cells and endothelium, double contours with new glomerular basement membrane formation underneath subendothelial deposits, extensive foot process effacement, and endocapillary proliferation (Figure 1B). These histopathological changes closely resemble those seen in human membranoproliferative glomerulonephritis (MPGN). Of note, glomerular injury was also observed in mice that did not develop tumors (#42 and #59) as well as in mice that received chemopreventive therapy and remained tumor-free (#78 and #221), suggesting that the MPGN-like glomerular changes are more likely attributable to VC itself rather than being an indirect effect of tumorigenesis.

### High dose EC treatment of rats induces dysregulation of complement regulators in the kidney, favoring activation of the alternative complement pathway

**FIGURE 2:**
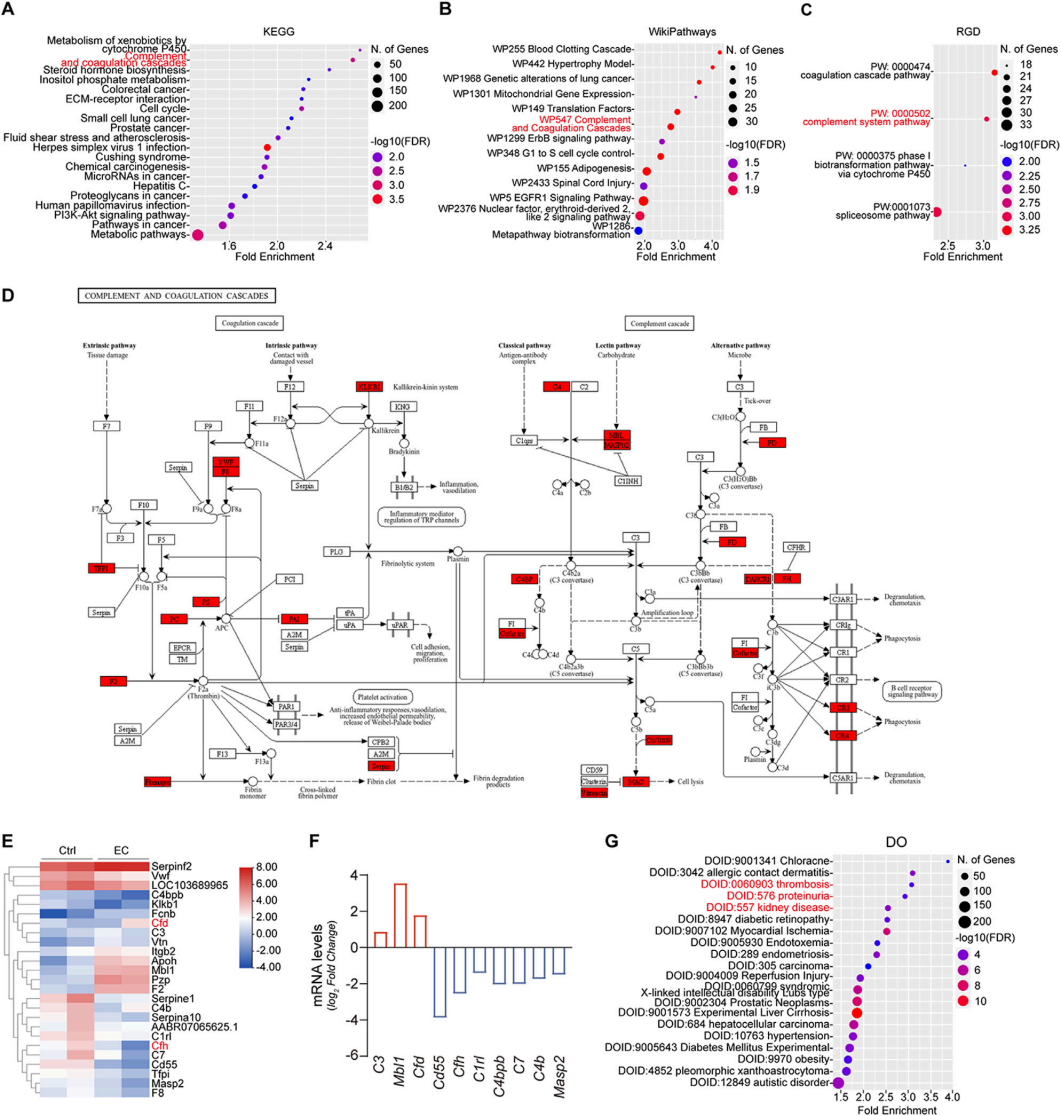
High dose EC treatment alters the expression of complement regulators, favoring a hyperactive alternative complement pathway. **(A)** Kyoto Encyclopedia of Genes and Genomes (KEGG), **(B)** WikiPathways, and **(C)** Rat Genome Database (RGD) pathway enrichment analyses of differentially expressed genes in renal transcriptome derived from high dose ethyl carbamate (EC)-treated rats versus normal control rats highlights the most affected pathways with significant statistical difference and high enrichment score. **(D)** The KEGG pathway map hsa04610 is involved in EC-induced kidney injury. The rectangle in red color indicates the EC-perturbed genes. **(E)** Heatmap representation and hierarchical cluster analysis of the differentially expressed genes enriched in complement pathway in kidneys from high dose EC-injured rats. Color scale represents the relative gene expression value for each gene using log_2_ median-centered intensity values. **(F)** Renal expression of mRNA levels of indicated genes in EC-treated rats and control rats (n = 2). **(G)** DEGs with statistical significance were subjected to enrichment analysis based on disease ontology (DO).

The effect of EC on rats had been investigated before (Wang et al., 2021). In that study, Sprague Dawley rats were subjected to intragastric gavage of 2 mL fermented wine containing a high concentration of EC (10 mg/kg) once a day for a week. Subsequently, renal transcriptome was profiled by RNA-sequencing. The data are publicly available (Wang et al., 2021) and were retrieved and analyzed. Shown in Figures 2A–C, to discover the pathways significantly altered by EC in the kidney, KEGG pathway enrichment analyses were performed on the differentially expressed genes (DEGs) with statistical difference and revealed that the complement and coagulation cascade is one of the leading pathways affected with significant statistical difference and high enrichment score. In consistency, WikiPathways enrichment also indicated that the WP547 Complement and Coagulation cascades is one of the leading pathways affected with significant statistical difference and high enrichment score. Similar findings were also made by Rat Genome Database (RGD) pathway enrichment analysis, which showed that the PW:0000502 complement system pathway is a top pathway affected by EC treatment with significant statistical difference and high enrichment score. The DEGs involved in the complement and coagulation cascades were further subjected to hierarchical cluster analysis and heat map representation (Figure 2E). Most of these DEGs are situated at key convergence points of regulating the complement activation pathways, as shown in the KEGG pathway map hsa04610 (Figure 2D). Among these, *CFD* and *CFH*, respectively key positive and negative regulators of the alternative pathway, were most perturbed. Specifically, *CFD* expression was upregulated 3.49-fold, while *CFH* expression was suppressed 5.9-fold. In addition, a number of other complement regulators or complement factors, including complement C3 (*C3*), mannose-binding lectin (protein A) 1 (*Mbl1*), CD55 molecule (*CD55*), *C1r1*, complement component 4 binding protein beta (*C4bpb*), *C7*, *C4b*, and mannose-binding lectin associated serine protease (*Masp*) 2, were also significantly altered by EC treatment (Figure 2F). Furthermore, to better understand the relationship between the affected genes and diseases, Disease Ontology (DO) analysis was performed. The leading DO enriched results with significant statistical difference and top enrichment scores can be divided into several categories separately related to different aspects of clinical features of MPGN (Sethi and Fervenza, 2012; Skerka et al., 2009), including DOID:0060903 thrombosis, DOID:576 proteinuria, DOID:557 kidney disease, and DOID:10763 hypertension as shown in Figure 2G. Collectively, these bioinformatics data suggest that EC treatment causes gene transcriptional changes favoring activation of the alternative complement pathway, and results in a molecular signature related to the disease traits of MPGN.

### The proactive effect of EC treatment on the alternative complement pathway is not present in the liver

**FIGURE 3:**
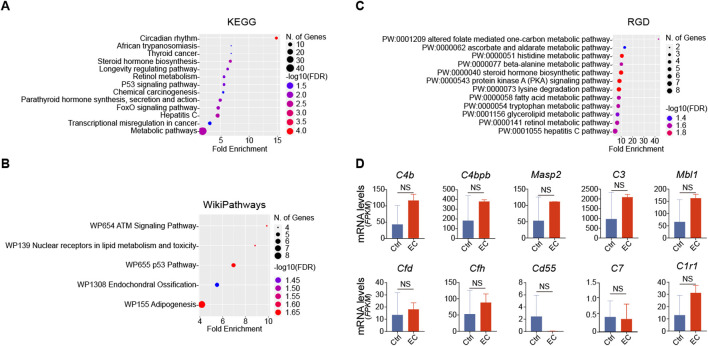
The effect of EC treatment on the complement pathway is unique to the kidney but not applicable to the liver. **(A)** Kyoto Encyclopedia of Genes and Genomes (KEGG), **(B)** WikiPathways, and **(C)** Rat Genome Database (RGD) pathway enrichment analyses of differentially expressed genes in RNA-sequencing transcriptome of livers derived from high dose EC-treated rats versus normal rats highlights the most affected pathways with significant statistical difference and high enrichment score. **(D)** Liver expression of mRNA levels (FPKM, fragments per kilobase of transcript per million fragments mapped) of complement factors or regulators in EC-treated rats and control rats. NS, non-significance (n = 2).

To test whether the effects of EC treatment observed in the kidney could be generalized to other organs, the liver RNA-sequencing transcriptome data were also retrieved from the Wang study (Wang et al., 2021) and analyzed. Shown in Figures 3A–C, pathway enrichment analyses, based on KEGG, WikiPathways and RGD, were performed on the DEGs in the liver between high EC-treated rats and control rats with statistical significance. All results indicated that none of the enriched pathways for the liver DEGs were related to the complement or coagulation cascades. In addition, RNA expression levels of *CFD*, *CFH* or other complement factors or regulators in the liver remained unchanged after EC treatment (Figure 3D). These data suggest that the effect of EC on the alternative complement pathway is specific to the kidney and does not apply to other organs, at least the liver.

### EC or VC treatment of mouse glomerular endothelial cells results in CFD induction and CFH suppression, associated with activation of the alternative complement pathway and cellular injury

**FIGURE 4:**
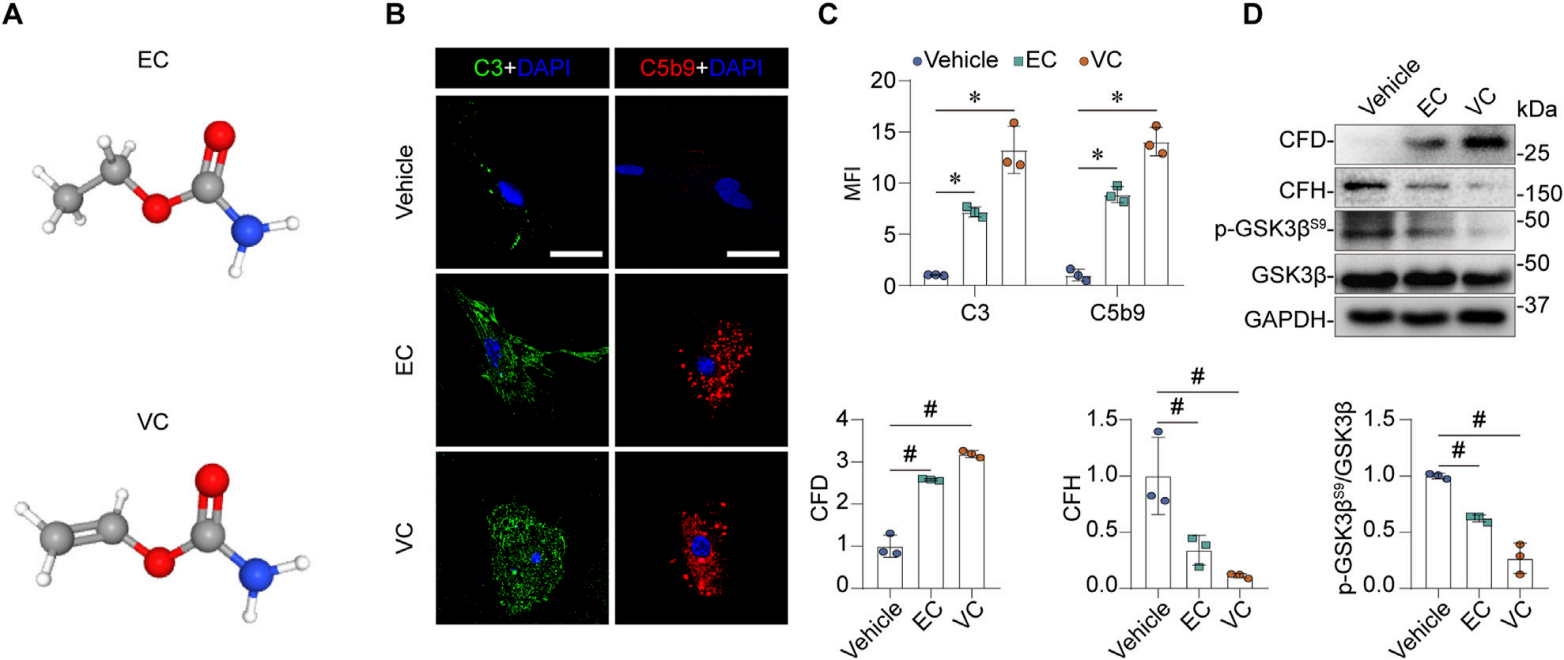
EC or VC activates the alternative complement pathway in cultured mouse glomerular endothelial cells, associated with CFD induction and CFH suppression. **(A)** The 3-D view of chemical structures of EC and VC. **(B–D)** Primary cultures of mouse glomerular endothelial cells were treated with EC, VC or vehicle for 24 h. **(B)** Cells were processed for fluorescent immunocytochemistry staining for C3 and C5b9, as shown by representative micrographs. Scale bar, 25 μm. **(C)** Arbitrary levels of mean fluorescence intensity (MFI) of C3 and C5b9 staining. *P < 0.05 (n = 3). **(D)** Cell lysates were subjected to immunoblot analysis for indicated proteins, followed by densitometry analysis. ^#^ *P* < 0.05 (n = 3).

The pathogenesis of MPGN predominantly involves glomerular endothelial cells (Cook and Pickering, 2015). To verify the above renal transcriptomic findings at protein expression levels and to validate if EC or VC is able to alter complement activation directly in glomerular cells, primary cultures of mouse glomerular endothelial cells were prepared and subjected to EC or VC stimulation for 24 h at a dose that had been confirmed to affect cellular signaling without impairing cell viability (Li et al., 2020; Cui et al., 2016). Since no glomerular deposition of immunoglobulin or microorganism infection was noted in the VC-treated mice or reported in the Wang study, DEGs related to the classical complement pathway or the lectin pathway in Figure 2F, including *Mbl1*, *C1r1*, *C4bpb*, *C4b* and *Masp2*, are thus unlikely involved in kidney injury. In addition, a number of publicly available single cell RNA-seq transcriptome of human or mouse kidneys suggest that the remaining DGEs in Figure 2F, including *C3*, *C7* and *CD55*, are predominantly expressed by kidney cells other than endothelial cells (Supplementary Figure 1). As such, we opted to focus on expression changes of the remaining *CFD* and *CFH* in cultured glomerular endothelial cells. Indeed, previous studies have implicated glomerular endothelial CFD and CFH in the pathogenesis of glomerular diseases (Chen et al., 2019; Wang et al., 2023). As a metabolite of EC, VC has a similar structure but features a vinyl group instead of an ethyl group (Figure 4A). Shown in Figures 4B–D, EC or VC treatment significantly induced CFD protein expression and repressed CFH protein expression, as revealed by western immunoblot analysis in conjunction with densitometric quantification. In parallel, complement activation was apparently triggered, as evidenced by fixation of C3 and C5b9 to cultured mouse glomerular endothelial cells shown by fluorescent immunocytochemistry staining. This resulted in glomerular endothelial injury, as evidenced by reduced inhibitory phosphorylation of GSK3β^S9^ thus denoting hyperactivity of GSK3β, a signaling mediator recently identified to play a key role in kidney injury (Liu and Gong, 2015).

### Ras signaling pathway is involved in EC and VC’s effects on complement activation

**FIGURE 5:**
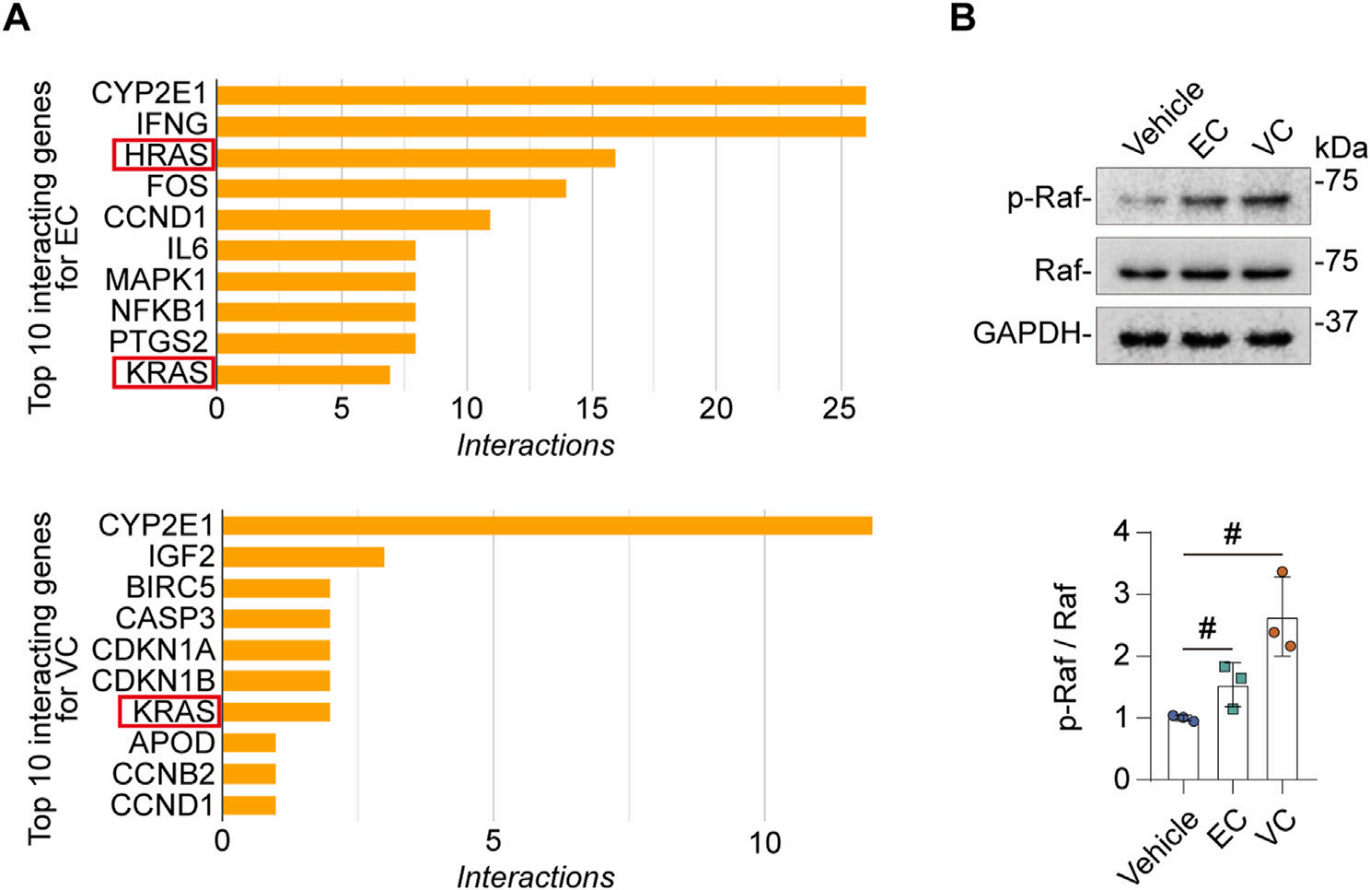
EC or VC injury activates Ras signaling pathway in mouse glomerular endothelial cells. **(A)** Top interacting genes/proteins for EC or VC were revealed by analyzing the Comparative Toxicogenomics Database and indicate that Ras is a common interacting gene/protein that interact with both EC and VC. **(B)** Primary cultures of mouse glomerular endothelial cells were treated with EC, VC or vehicle for 24 h. Cell lysates were subjected to immunoblot analysis for indicated proteins, followed by densitometry analysis. ^#^ *P* < 0.05 (n = 3).

**FIGURE 6:**
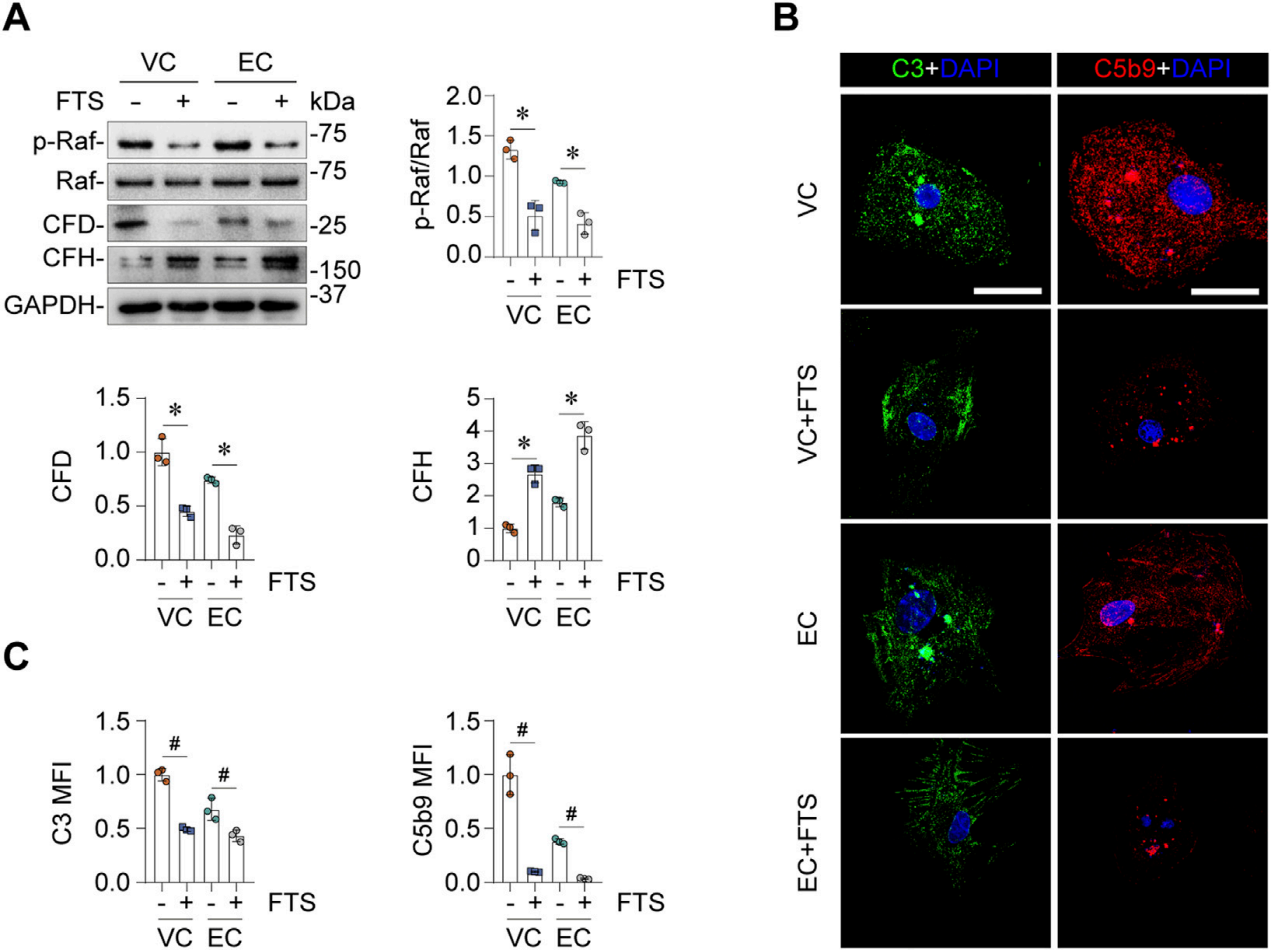
Ras signaling mediates the activating effects of EV or VC on alternative complement pathway. Primary cultures of mouse glomerular endothelial cells were treated with EC or VC for 24 h in the presence or absence of farnesyl thiosalicylic acid (FTS), a synthetic small molecule inhibitor of Ras. **(A)** Cell lysates were subjected to immunoblot analysis for indicated proteins, followed by densitometry analysis. ^*^ *P* < 0.05 (n = 3). **(B)** Cells were processed for fluorescent immunocytochemistry staining for C3 and C5b9, as shown by representative micrographs. Scale bar, 25 μm. **(C)** Arbitrary levels of mean fluorescence intensity (MFI) of C3 and C5b9 staining. ^#^ *P* < 0.05 (n = 3).

Next, we sought to determine the molecular mechanisms responsible for EC- or VC-induced complement activation in glomerular endothelial cells. The Comparative Toxicogenomics Database was utilized to acquire the curated information about chemical-gene/protein interactions based on published literature. Shown in Figure 5A, cytochrome P450 family 2 subfamily E member 1 (*CYP2E1*), interferon gamma (*INFG*), HRas proto-oncogene (*HRAS*), Fos proto-oncogene (*FOS*), cyclin (*CCN*) *D1*, interleukin 6 (*IL6*), mitogen-activated protein kinase 1 (*MAPK1*), nuclear factor kappa B subunit 1 (*NFKB1*), prostaglandin-endoperoxide synthase 2 (*PTGS2*), *KRAS* are the top interacting genes for EC, while the top interacting genes for VC include *CYP2E1*, insulin like growth factor 2 (*IGF2*), baculoviral IAP repeat containing 5 (*BIRC5*), caspase 3 (*CASP3*), cyclin dependent kinase inhibitor (*CDKN*) *1A*, *CDKN1B*, *KRAS*, apolipoprotein D (*APOD*), *CCNB2*, *CCND1*. Thus, the top interacting genes shared by both EC and VC are *CYP2E1*, *CCND1* and *RAS*. To understand how these genes are involved in the biological actions of EC and VC, related literature was reviewed. To this end, previous studies have shown that the cytochrome P-450 enzyme *CYP2E1* is involved in the oxidation of both EC and VC (Forkert, 2010), and that *CCND1* or cyclin D1 induction is a mechanism shared by EC and VC for their proliferative effects in tumorigenesis (Mori et al., 2001; Alyaqoub et al., 2008). Since there is no literature directly supporting the involvement of *CYP2E1* or *CCND1* in regulating complement activation, we therefore opted to focus on the signaling pathway transduced by the RAS protein. To examine if EC or VC actually affects the signaling activity of the Ras pathway, cell lysates of mouse glomerular endothelial cells were subjected to immunoblot analysis after VC or EC injury. Shown in Figure 5B, EC or VC treatment triggered phosphorylation of Raf, a signaling mediator immediately downstream of Ras, suggesting an enhanced Ras signaling. To further test if this induced Ras signaling mediates EC- or VC-triggered complement activation, mouse glomerular endothelial cells were stimulated with EC or VC in the presence or absence of farnesyl thiosalicylic acid (FTS), a Ras inhibitor (Rotblat et al., 2008). Shown in Figures 6A–C by immunoblot analysis, FTS co-treatment, as expected, abolished Raf phosphorylation triggered by EC or VC, consistent with its Ras inhibitory property. This was associated with abrogated CFD induction and mitigated CFH repression upon EC or VC exposure. Consequently, fixation of C3 and C5b9 on EC- or VC-injured mouse glomerular endothelial cells was also abolished by FTS co-treatment, suggesting that Ras signaling is required for EC or VC-triggered complement activation.

## Discussion

MPGN is a type of glomerulonephritis characterized by deposits in glomerular mesangium and basement membrane, which activate the complement system and damage the glomeruli (Sethi and Fervenza, 2012). Histopathologically, MPGN is featured by a pattern of glomerular injury defined by mesangial and endocapillary hypercellularity, mesangial matrix expansion, and double contouring of glomerular basement membrane (Fogo et al., 2015). Although the pathomorphology of MPGN has been well established for decades, its pathogenesis is still elusive, and disease mechanism-driven treatments are lacking (Noris et al., 2023). Much of this delay is largely due to the limited availability of useful preclinical models of MPGN (Vernon et al., 2011). The present study demonstrated that VC-treated mice developed glomerular injury recapitulating the pathology of human MPGN. To the best of our knowledge, this is the first report of a drug-induced animal model of human MPGN.

Human MPGN is classified into three subtypes based on histopathological differences (Fogo et al., 2015; Noris et al., 2023). Both type I and type III MPGN are immune complex-mediated glomerulonephritis associated with immune complex deposition in glomeruli, and glomerular injury is believed to be mediated by complements activated by immune complexes. Type I MPGN, most commonly found in clinical practice, is associated with subendothelial and mesangial immune complex accumulation, while type III MPGN is rare, involving subepithelial and subendothelial immune complex deposits. In contrast, type II MPGN is associated with scarce immune complex deposition in glomeruli but caused by aberrant activation of the complement alternative pathway. Under EM, type II MPGN is characterized by highly osmiophilic dense deposits within the lamina densa of the glomerular basement membrane, which is identified by immunoflurescent staining to comprise isolated or predominant C3 (Fogo et al., 2015). In our VC-induced mouse model of glomerular injury, immunoglobulin staining was negative, suggesting the glomerular lesion in our model is highly akin to type II MPGN rather than type I or III MPGN. Type II MPGN is also known as dense deposit disease (DDD). In typical DDD, when viewed under the electron microscope, continuous, characteristic dense ribbon-like deposits are found along the basement membranes of the glomeruli, tubules, and Bowman’s capsule (Fogo et al., 2015). However, in our VC-injured mice, isolated granular C3 staining was noted in mesangium along with coarse linear capillary wall staining, suggesting that this model does not resemble a typical DDD. Of note, based on electron microscopic findings, type II MPGN forms a continuum with C3 glomerulonephritis, which is characterized by amorphous C3 deposition within the capillary wall and mesangium (Cook and Pickering, 2015). Typical type II MPGN or DDD and C3 glomerulonephritis now constitutes the two subgroups of C3 glomerulopathy. To this end, our VC-induced mouse model developed MPGN with glomerular capillary wall deposition of granular C3. This pathologic pattern of glomerular injury is more akin to C3 glomerulonephritis. The fact that our model did not show a characteristic pattern of type II MPGN suggests that more studies need to be done in experimental models to identify other subclasses of MPGN.

This VC-elicited MPGN model is unique because previous models of MPGN are very few and largely confined to genetically engineered animals with deficiency of complements or complement regulators (Vernon et al., 2011). For instance, dogs and sheep with C3 deficiency developed spontaneous type I MPGN (Vernon et al., 2011; Cork et al., 1991). In addition, genetically engineered mice overexpressing thymic stromal lymphopoietin had mixed cryoglobulinaemia and subsequently sustained MPGN type I (Taneda et al., 2001). Moreover, pigs with a genetic deficiency of CFH spontaneously developed glomerulonephritis before birth, resembling MPGN type II (Hogasen et al., 1995). Similarly, mice genetically engineered with a deficiency of CFH also developed MPGN with glomerular capillary wall C3 deposition (Pickering et al., 2002). While these very few genetic models may resemble human MPGN to some extent, their etiology is restricted to a single gene disorder, which is very rare for patients with MPGN. Thus, findings from genetically engineered animal models with global deficiency of a specific gene may be incomplete and limit our understanding of the true pathogenic mechanisms of common MPGN. In contrast, based on kidney injury induced by VC or EC, which are naturally occurring compounds in fermented foods or beverages (Gowd et al., 2018), our model may mirror a more natural disease course of human MPGN. Furthermore, genetically engineered animal models are much more expensive, whereas our drug-based model would have a significant cost advantage.

How does VC or EC induce complement activation in glomerular cells and thereby cause MPGN-like glomerular injury? While some other signaling pathways may also be involved, our mechanistic studies suggest that Ras signaling is activated by EC or VC in glomerular endothelial cells and is required for EC or VC-induced CFD upregulation and CFH repression. This result is consistent with previous findings that hyperactivity of Ras signaling pathway and K-ras mutations are commonly detected in VC- or EC-elicited tumors in a number of organs like the lung (Massey et al., 1995). In addition, Ras signaling has been reported as a key regulatory mechanism of complement activation. For instance, ovarian surface epithelial cells from mice with conditional K-ras^G12D^ gain of function mutation exhibited complement activation, which was attributed to intrinsic upregulation of CFD and repression of CFH (Suryawanshi et al., 2014), a pattern of changes in complement regulators identical to what was observed in our model. This effect was abrogated by small molecule inhibitors that blocks Kras pathway (Suryawanshi et al., 2014). In agreement, the effect of EC or VC on complement activation in our model was also abolished by FTS, a Ras inhibitor. In our study, VC seems to be more potent than EC in activating the complement pathway and conceivably in inducing MPGN-like kidney injury. This may be attributable to the chemostructural difference between EC and VC. EC contains an ethyl group (CH_3_-CH_2_-) that undergoes enzymatic oxidation to form a vinyl group (CH_2_ = CH-), thereby generating VC. As an intermediate metabolite of EC, VC undergoes epoxidation to form a highly electrophilic vinyl carbamate epoxide, which can covalently bind to macromolecules and exert biological effects. This increased reactivity makes VC more biologically active than EC (Dahl et al., 1978), in terms of carcinogenesis or complement activation as shown here. It is plausible that the covalent binding of electrophilic reactants like vinyl carbamate epoxide to cellular signaling proteins like Ras in kidney cells may activate the Ras pathway and subsequently mediate complement activation in the kidney.

**FIGURE 7:**
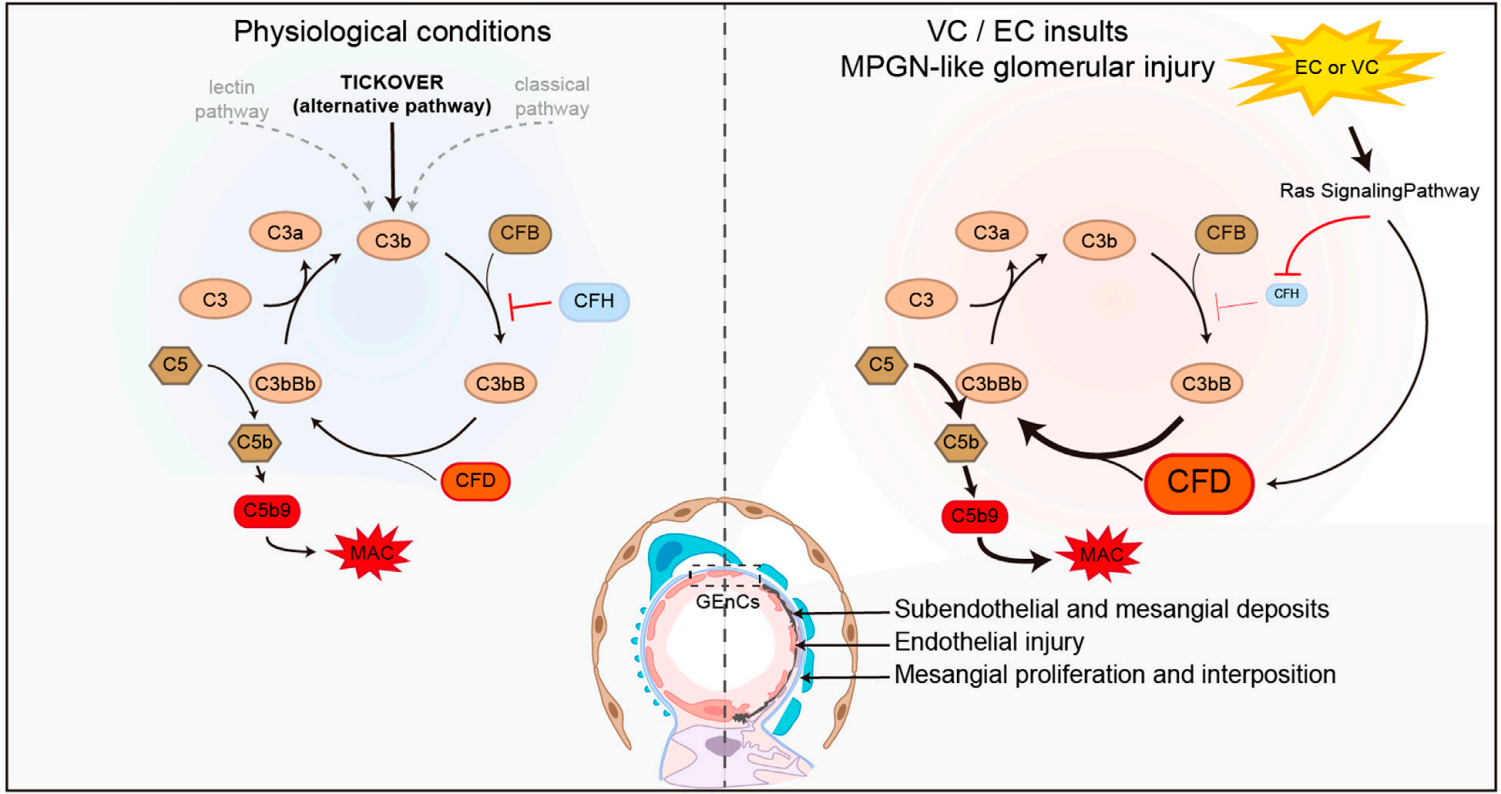
A hypothetical working model possibly operative in the mouse model of VC-induced MPGN. Under physiological conditions (left panel), the alternative complement pathway is constantly activated through a slow spontaneous “tickover” mechanism. Complement factor (CF) D and H are critical positive and negative regulators of the C3 “tickover.” VC or EC stimulation (right panel) of the glomerular endothelial cells (GEnCs) activates Ras signaling, which subsequently, via unknown mechanisms, augments the expression of CFD, and suppress the expression of CFH, resulting in a net effect favoring activation of the alternative complement pathway and formation of the membrane attack complex (MAC). Continuous complement fixation causes MPGN-like glomerular injury and changes, including subendothelial and mesangial deposits, endothelial injury, new glomerular basement membrane (GBM) formation, endocapillary and mesangial proliferation.

Based on our data and previous findings, the following hypothetical working model is posited to be operative in our mouse model of MPGN (Figure 7). Under physiological conditions, it is known that the alternative complement pathway is constantly activated through a slow spontaneous “tickover” mechanism driven by spontaneous hydrolysis of an internal thioester bond in C3 (Bexborn et al., 2008; Peters, 2023). CFD is an early and essential regulator facilitating this process (Barratt and Weitz, 2021), while CFH is critical for the negative modulation of C3 “tickover” (Ferreira et al., 2010). VC or EC stimulation of the glomerular endothelial cells activates Ras signaling, which subsequently, via unknown mechanisms, augments the expression of CFD, and suppresses the expression of CFH, resulting in a net effect favoring activation of the alternative complement pathway. Continuous complement fixation causes subendothelial and mesangial deposits in glomeruli and damages endothelial cells. This results in endothelial repair and regrowth, new GBM formation, and inflammatory response and the ensuing endocapillary and mesangial proliferation, culminating in glomerular lesions reminiscent of human MPGN (Sethi and Fervenza, 2012; Fervenza et al., 2012).

In addition to the complement pathway, other biological pathways altered in the kidney of EC-treated animals may also contribute to MPGN-like kidney injury. For example, the Blood Clotting Cascade and Coagulation Cascade are among the top pathways affected by EC treatment, showing significant statistical difference and high enrichment score, as evidenced by KEGG, WikiPathways and RGD pathway enrichment analyses. Consistently, Disease Ontology (DO) analysis also indicates that DOID:0060903 thrombosis is a highly enriched disease feature and etiological factor, which is also linked to the clinicopathological characteristics of MPGN. Indeed, thrombotic microangiopathy, featured by endothelial damage and microvascular thrombosis, has been shown to overlap or associate with C3 glomerulopathy and MPGN, highlighting a common pathogenic pathway (Ravindran et al., 2023; Timmermans et al., 2023; Herzenberg et al., 1998). Mechanistically, in addition to the Ras signaling pathway, other signaling pathways may also contribute to EC/VC-induced glomerular endothelial injury and complement activation. For instance, EC/VC has been shown to activate the NFκB pathway (Stathopoulos et al., 2007; Kassie et al., 2010), which has been centrally involved in endothelial cell activation, thrombosis, and complement-mediated renal injury (Mussbacher et al., 2019; Liu et al., 2018). Our study is limited by several factors. First, the histology-based MPGN-like glomerular injury should be corroborated with additional functional data, such as serum creatinine, blood urea nitrogen and urinary protein levels. In addition, direct measurement of complement activity in either the kidney or the blood was not conducted. Future in-depth studies are needed to validate our findings, further characterize this VC-elicited MPGN mouse model, and develop specific target-based novel therapies in glomerular etiology of MPGN.

In summary, VC, a metabolite of EC, caused glomerular injury and MPGN-like pathological changes with characteristic electron microscopic lesions as well as isolated glomerular C3 deposition in glomerular capillary wall and mesangium. VC exerted this pathogenic effect likely through Ras-mediated CFD overexpression and CFH repression, leading to activation of the alternative complement. This model will be an important tool instrumental for studying the pathogenesis and novel therapies of MPGN.

## Data Availability

The original contributions presented in the study are included in the article/Supplementary Material, further inquiries can be directed to the corresponding author.
