# Supplementary material for: Glomerular injury induced by vinyl carbamate in A/J inbred mice: a novel model of membranoproliferative glomerulonephritis
Source: Front Pharmacol. 2024 Sep 6;15:1462936. doi: 10.3389/fphar.2024.1462936 (PMC11412833; doi:10.3389/fphar.2024.1462936)
Supplement: Supplementary file 1 [file DataSheet1.PDF]

Supplemental Table 1. Histopathologic evaluation of moribund mice after vinyl carbamate treatment.

Animal #	Time at sacrifice	Treatment	Microscopic tumor#	Liver histology	Kidney histology
**Normal control**					
2	6 wks	Control	0	Normal	Normal
**VC-5wks**					
32	5 wks	VC	1	Normal	MPGN
42	5 wks	VC	0	Normal	MPGN
59	5 wks	VC	0	Normal	MPGN
**VC-6wks**					
30	6 wks	VC	1	Normal	MPGN
33	6 wks	VC	2	Normal	MPGN
178	6 wks	VC	3	Normal	MPGN
**VC-8wks**					
17	8 wks	VC	3	ND	MPGN
23	8 wks	VC	8	ND	MPGN
28	8 wks	VC	5	ND	MPGN
43	8 wks	VC	4	ND	MPGN
51	8 wks	VC	9	ND	Resolved MPGN?
57	8 wks	VC	2	ND	Resolved MPGN?
**Chemoprevention**					
78	6 wks	VC +myo-inositol	0	Normal	MPGN
221	6 wks	VC + Dexamethasone	0	Normal	MPGN

Abbreviations: MPGN, Membranoproliferative glomerulonephritis; ND, not detected; VC, vinyl carbamate; wks, weeks after vinyl carbamate injection

Supplemental Figure 1

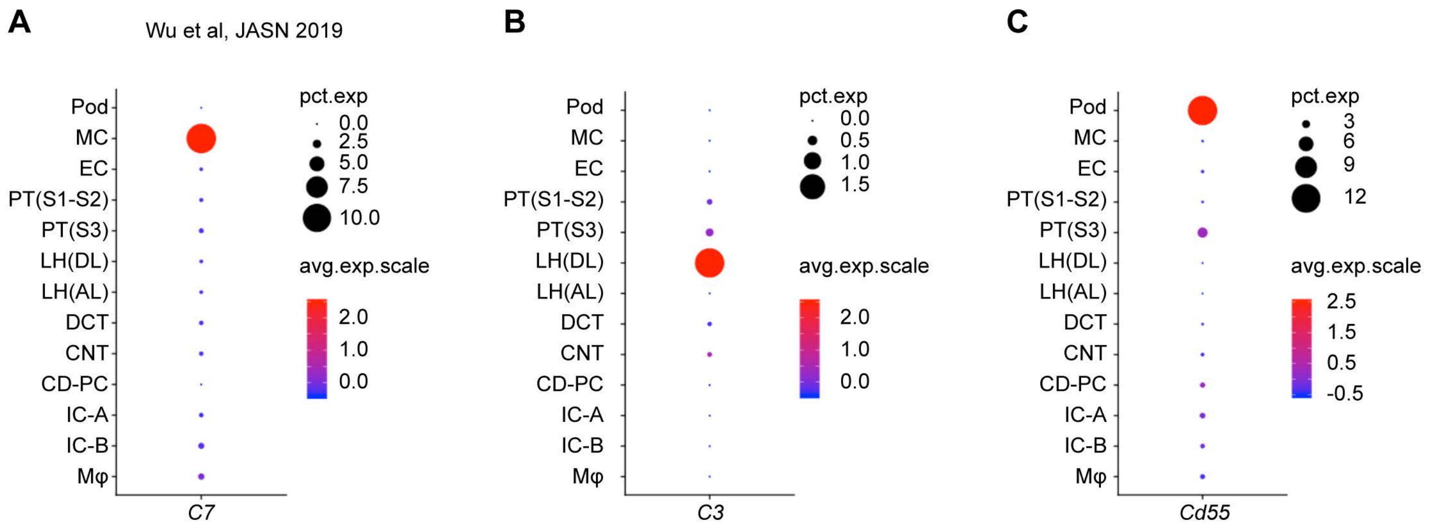

Supplemental Figure 1. *C3*, *C7,* and *CD55* are expressed predominantly in other renal cell types but minimally in renal endothelial cells. A *post hoc* analysis was performed on the single nucleus RNA-sequencing (snRNAseq) transcriptome of mouse kidneys based on the Wu Healthy Mouse Dataset that is publicly-available from Kidney Interactive Transcriptomics [https://humphreyslab.com/SingleCell/]. The distribution and relative levels of mRNA expression of (A) *C7*, (B) *C3*, and (C) *CD55* are shown. Abbreviations: Pod, podocyte; MC, mesangial cell; EC, endothelial cell; PT(S1-S2), proximal tubular(Segment 1-2); PT(S3), proximal tubular(Segment 3); LH(DL), loop of henle(descending limb); LH(AL), loop of henle(ascending limb); DCT, distal convoluted tubule; CNT, connecting tubule; CD-PC, collecting duct principal cell; IC-A, alpha intercalated cell; IC-B, beta intercalated cell; Mφ, macrophage.
